# Inducing perylenequinone production from a bambusicolous fungus *Shiraia* sp. S9 through co-culture with a fruiting body-associated bacterium *Pseudomonas fulva* SB1

**DOI:** 10.1186/s12934-019-1170-5

**Published:** 2019-07-05

**Authors:** Yan Jun Ma, Li Ping Zheng, Jian Wen Wang

**Affiliations:** 1 College of Pharmaceutical Sciences, Soochow University, Suzhou, 215123 China; 2 Department of Horticultural Sciences, Soochow University, Suzhou, 215123 China

**Keywords:** *Shiraia*, Fruiting body, Associated bacteria, Co-culture, Perylenequinones, Hypocrellin A

## Abstract

**Background:**

Fungal perylenequinonoid (PQ) pigments from *Shiraia* fruiting body have been well known as excellent photosensitizers for medical and agricultural uses. The fruiting bodies are colonized by a diverse bacterial community of unknown function. We screened the companion bacteria from the fruiting body of *Shiraia* sp. S9 and explored the bacterial elicitation on fungal PQ production.

**Results:**

A bacterium *Pseudomonas fulva* SB1 isolated from the fruiting body was found to stimulate the production of fungal PQs including hypocrellins A, C (HA and HC), and elsinochromes A–C (EA, EB and EC). After 2 days of co-cultures, *Shiraia* mycelium cultures presented the highest production of HA (325.87 mg/L), about 3.20-fold of that in axenic culture. The co-culture resulted in the induction of fungal conidiation and the formation of more compact fungal pellets. Furthermore, the bacterial treatment up-regulated the expression of polyketide synthase gene (*PKS*), and activated transporter genes of ATP-binding cassette (*ABC*) and major facilitator superfamily transporter (*MFS*) for PQ exudation.

**Conclusions:**

We have established a bacterial co-culture with a host *Shiraia* fungus to induce PQ biosynthesis. Our results provide a basis for understanding bacterial–fungal interaction in fruiting bodies and a practical co-culture process to enhance PQ production for photodynamic therapy medicine.

**Electronic supplementary material:**

The online version of this article (10.1186/s12934-019-1170-5) contains supplementary material, which is available to authorized users.

## Background

Perylenequinones (PQs) comprise a family of natural pigments characterized by 3,10-dihydroxy-4,9-perylenequinone chromophore, which are being explored as a group of reactive oxygen species (ROS)-generating photosensitizers for medical and agricultural uses [1]. The PQ-rich fruiting bodies of a bambusicolous fungus *Shiraia bambusicola* have been used in traditional Chinese medicine for treating vitiligo, stomachache, psoriasis and rheumatic pain [2]. The PQ pigments including hypocrellin A–D (HA, HB, HC and HD), elsinochrome A–C (EA, EB and EC), shiraiachrome A–C (SA, SB and SC) have attracted intense interest as promising photosensitizers for photodynamic therapy (PDT) and antimicrobial agents [3–6]. Due to the complexity and difficulty in their chemical synthesis [7], the fruiting body is still the traditional main resource for PQ supply [8, 9]. Recently, *Shiraia* mycelium cultures have been applied intensively as a promising alternative for PQ production [10]. However, the major obstruction for a large-scale production of PQs is the lower yields either in solid-state fermentation (2.02 mg/g DW for HA) [8] or in liquid fermentation (approximately 10–40 mg/L for HA [11] and 9–74 mg/L for elsinochromes [12]). Even with rich carbon and nitrogen supply in medium, no hypocrellins were produced in the submerged cultures of *Shiraia* sp. SUPERH-168 [13] or *S. bambusicola* S8 [14]. Thus, the induction and improvement of PQ production in *Shiraia* mycelium cultures is needed.

Recently, microorganism co-culture inspired by the natural microbe communities is becoming one of OSMAC (One Strain, Many Compounds) strategies to increase microbial chemodiversity [15–17]. Co-cultivation of fungi with bacteria is becoming a powerful tool for either inducing silent gene clusters for new metabolites and/or enhancing the production of present compounds in low yield. The co-cultivated microorganisms are selected from soil, water, animals or plants so as to be capable of successfully competing for limited resources and antagonism in the mimic complex habitats [18]. Although fruiting bodies harbor diverse bacteria with influence on fungal growth, fruit body formation and aroma [19–21], little information is available regarding the co-cultivated microorganisms from fruiting bodies. To understand the interactions between hosting fungi and bacteria in the fruiting body, and establish a fungal–bacterial co-culture process for eliciting target PQ compounds, we continued our work on PQ-rich *Shiraia* fruiting body [22]. An effective PQ-promoting bacterial isolate SB1 was selected for the co-culture for the enhanced HA production in submerged cultures. Furthermore, we investigated the interaction between the bacterium and *Shiraia* sp. S9. Our findings could be used for developing novel eliciting method for PQ production by mycelium cultures and also for understanding fungal–bacterial interactions in a fruiting body.

## Results

### The screening of the associated bacteria

**Fig. 1:**
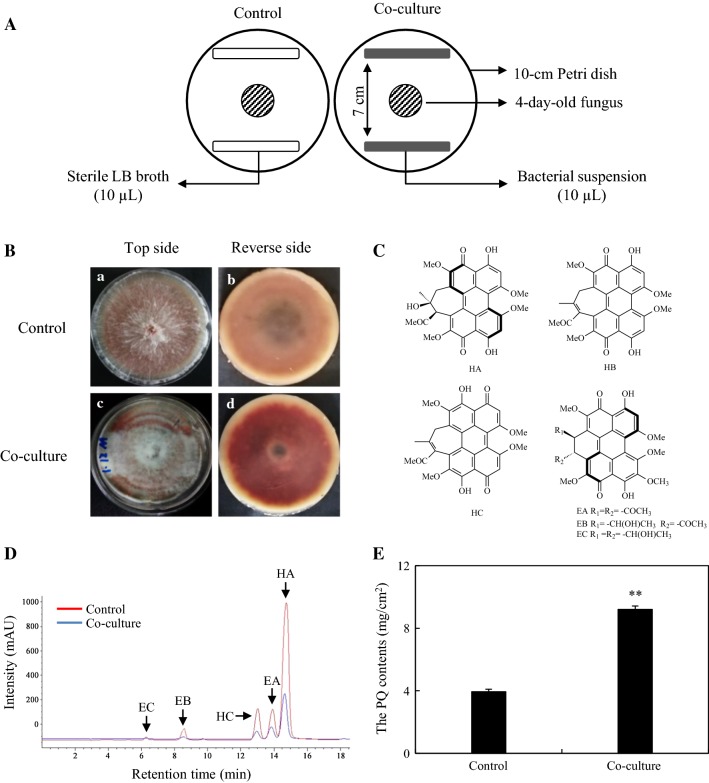
Effects of live SB1 on the fungal growth and PQ accumulation in solid cultures of *Shiraia* sp. S9 in PDA plate. **A** Scheme of the in vitro confrontation assay. **B** The effects of live SB1 on the growth and red pigments secretion of S9. **C** Structures of individual PQs. **D** The chromatogram of enhanced individual PQs in mycelium treated with live SB1. **E** The total PQ contents of S9 in PDA plate. Values are mean ± SD from three independent experiments. **p < 0.01 versus control group

A total of 31 bacterial isolates were obtained from the fresh fruiting body of *S. bambusicola* (Additional file 1: Table S1). To investigate the role of those bacteria on the PQ accumulation of host fungus *Shiraia* sp. S9 in PDA plate, a vitro fungal–bacterial confrontation assay was carried out (Fig. 1A). In the confrontation assay, the bacterial isolate No. 11 (named SB1) exhibited a significant capacity to stimulate the fungal accumulation of red PQ pigments in the plates (Additional file 1: Figure S1 and Table S1). As shown in Fig. 1, axenic *Shiraia* sp. S9 presented abundant aerial and substrate mycelia with red PQ pigments in the plate (control in Fig. 1B). The live bacterium stimulated the secretion of red pigments into medium from the fungus with sparse white aerial mycelia, and the reverse side of the colony has the deep red pigment (co-culture in Fig. 1B). All of individual PQs including HA, HC, EA and EB were stimulated under the treatment of live SB1 (Fig. 1D and Table 1). The presence of SB1 even induced a slight but significant increase of EC (0.19 mg/cm^2^), whereas HB and EC were not detected from this strain in the control (Table 1). The total PQ accumulation in PDA plate was enhanced to 9.21 mg/cm^2^, a 2.34-fold of that in control group (Fig. 1E).

**Table 1:** Effects of live bacterium *P. fulva* SB1 (No. 11) on the individual PQ contents in PDA plates and submerged cultures of *Shiraia* sp. S9

Cultural method	HA	HC	EA	EB	EC
Individual PQ contents in PDA plate (mg/cm^2^)					
Control	2.96 ± 0.14	0.31 ± 0.01	0.49 ± 0.01	0.18 ± 0.01	ND
Co-culture	6.18 ± 0.05**	1.32 ± 0.12**	1.08 ± 0.02*	0.44 ± 0.01*	0.19 ± 0.02**
Intracellular PQs in submerged culture (mg/g DW)					
Control	5.33 ± 0.54	1.88 ± 0.13	1.07 ± 0.02	0.26 ± 0.01	0.31 ± 0.01
Co-culture	8.55 ± 1.14**	3.06 ± 0.06*	2.08 ± 0.04*	3.14 ± 0.10**	3.21 ± 0.26**
Extracellular PQs in cultural culture (mg/L)					
Control	1.03 ± 0.01	0.71 ± 0.11	0.28 ± 0.03	ND	ND
Co-culture	1.64 ± 0.13*	1.09 ± 0.17*	0.48 ± 0.02*	0.29 ± 0.01**	0.14 ± 0.01*

**Fig. 2:**
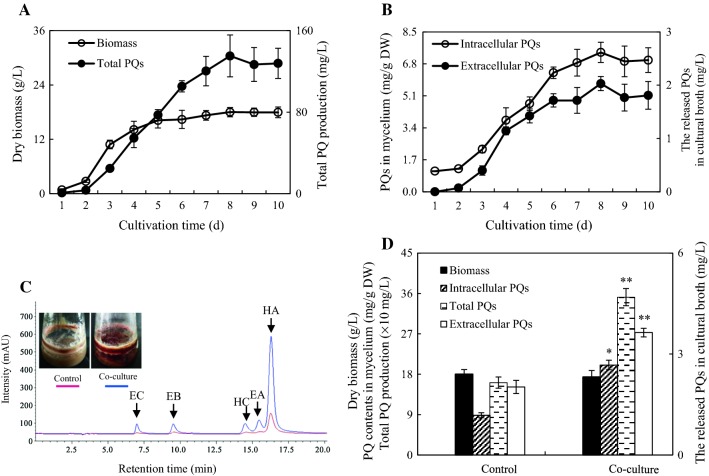
Effects of live SB1 on the fungal growth and PQ accumulation in submerged cultures of *Shiraia* sp. S9. **A** Time profiles of hyphal biomass and total PQ production. **B** Time profiles of PQs in mycelium and the released PQs in broth. **C** The chromatogram of enhanced individual PQs in mycelium of submerged fermentation of S9 treated with *P. fulva* SB1 cells. **D** The effects of live SB1 on fungal growth, intracellular PQ, extracellular PQ and total PQ production. Total PQ production refers to the sum of the intracellular and extracellular PQs. Values are mean ± SD from three independent experiments. *p < 0.05, **p < 0.01 versus control group

The procedure of fungal–bacterial confrontation assay and co-culture was the same as specified in Figs. 1 and 2, respectively. Values are mean ± SD from three independent experiments

*ND* Not detected

* *p* < 0.05, ** *p* < 0.01 versus control group

In an axenic mycelium culture, *Shiraia* sp. S9 has a typical time courses of hypha growth and PQ production (Fig. 2). The hypha biomass showed an exponential growth from day 1–5 and reached the highest (18.01 g/L) on day 8 (Fig. 2A). The PQs in mycelium (intracellular PQs) contents initially maintained a small quantity (1.10–1.23 mg/g DW) within 2 days, then increased with time up to day 8 with the maximum (7.41 mg/g DW) (Fig. 2B). The PQs were released in the medium as extracellular PQs with a pattern close to that in mycelium (Fig. 2B), while the total production of PQs increased to 135.13 mg/L on day 8 (Fig. 2A). In a liquid co-culture of fungus S9 with live SB1, the individual PQ production was enhanced markedly (Table 1 and Fig. 2C). Although the fungal biomass was not altered obviously, the live SB1 promoted total PQ production to 351.65 mg/L on day 8, a 2.18-fold of that in the control group (Fig. 2D). It was noteworthy that SB1 stimulated not only intracellular PQs in mycelium (2.26-fold), but also the extracellular PQs released in medium (1.80-fold) (Fig. 2D). Furthermore, both EB and EC were induced into the medium only under the SB1 treatment (Table 1).

### Identification and characterization of SB1 strain

**Fig. 3:**
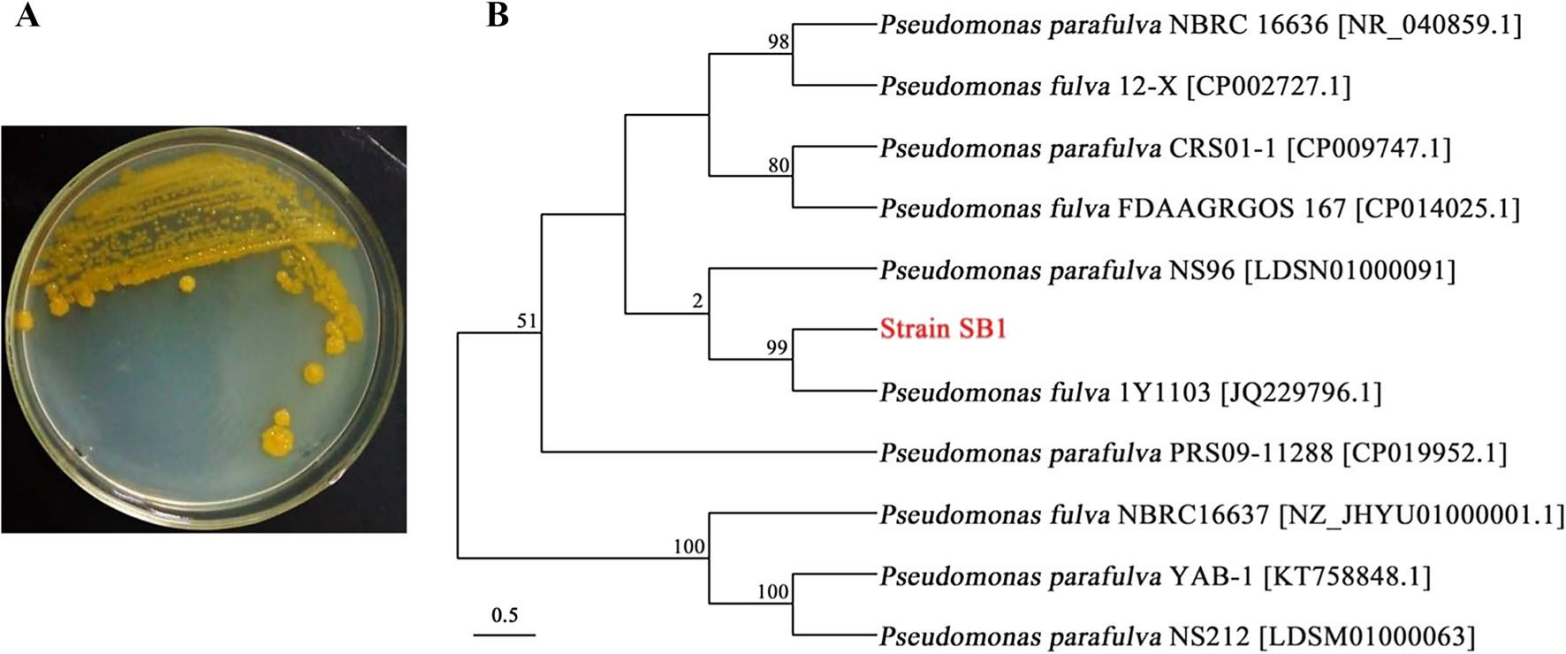
**A** Macroscopic colony appearance of SB1 strain on LB agar plate for 24 h. **B** The phylogenetic tree using 16S rDNA gene sequences showing the relationship between SB1 and other *Pseudomonas* species. Numbers at the nodes indicated bootstrap values from the neighborhood-joining analysis of 1000 replications. Scale bar indicated nucleotide substitutions per nucleotide position

The SB1 colony appeared round, smooth, flat to convex in shape and creamy yellow in color (Fig. 3A). A length of 1075 bp sequence was amplified from the genome DNA of strain SB1 with the 27F/1492R as primers and the sequence has been deposited in GenBank under the accession number MF058662.1. It was initially classified as *Pseudomonas fulva* based on hit taxon strain with the 16S rDNA sequence data in the BLAST server (https://blast.ncbi.nlm.nih.gov/Blast.cgi) compared to *P. fulva* 1Y1103 (GenBank accession number, JQ229796.1) with a similarity of 99%. A phylogenetic tree of *P. fulva* SB1 based on 16S rDNA sequences was built by using MEGA software (version 7.0.26) and neighbor-joining method (Fig. 3B). Additional file 1: Table S2 showed some biochemical characteristics of *P. fulva* SB1 as an aerobic Gram-negative bacterium.

### Elicitation on fungal PQ production by the bacterium

**Fig. 4:**
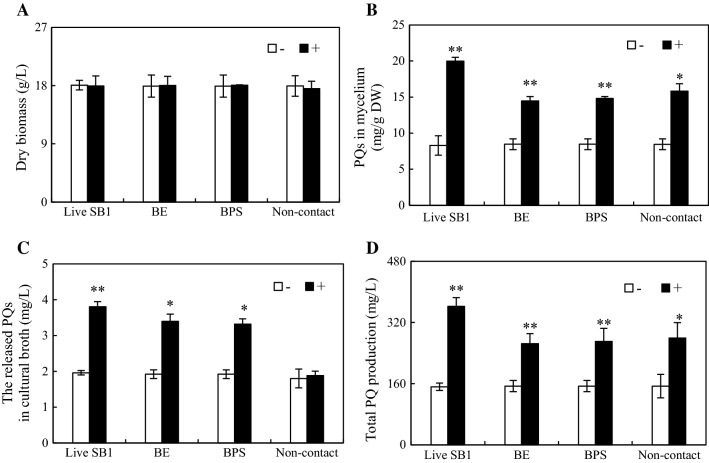
Effects of live *P. fulva* SB1 cells, hot water extract (BE), crude polysaccharide (BPS) and bacteria in the dialysis tubing on mycelium dry biomass (**A**), PQ contents in mycelium (**B**), the released PQs in cultural broth (**C**) and total PQ production (**D**) in submerged culture of *Shiraia* sp. S9. The S9 strain treated with the equal volumes of sterile LB broth or water instead of live SB1 suspension or bacterial extracts solution were used as control groups, respectively. ‘Non-contact’ indicates live bacteria in dialysis tubing treatment. ‘−’ indicates control groups. ‘+’ indicates different treatment groups. Values are mean ± SD from three independent experiments. *p < 0.05, **p < 0.01 versus control groups

To investigate the elicitation of SB1 on fungal growth and PQ accumulation, the live bacteria, the bacterial water extracts (BE) and crude polysaccharides (BPS), and bacteria in the dialysis tubing (non-contact) were applied to *Shiraia* sp. S9 cultures, respectively (Fig. 4). The BE at 200 mg/L and BPS at 100 mg/L were selected as active elicitors on the basis of the results from the concentration study (Additional file 1: Figures S2 and S3). After the elicitation treatments including live SB1, extracts (BE and BPS) or bacteria in the dialysis tubing, there was no obvious alters in fungal biomass (Fig. 4A). The treatment of live SB1, BE and BPS exhibited the capacity to stimulate PQ biosynthesis and release in the culture medium (Fig. 4B, C). SB1 in the dialysis tubing (non-contact) promoted the biosynthesis of intracellular PQs, but had no effects on PQ excretion (Fig. 4B). It was noteworthy that live SB1 stimulated not only intracellular PQs by 141.2% to 19.99 mg/g DW, but also PQ accumulation in medium by 94.0% to 3.80 mg/L (Fig. 4B, C), leading to the total PQ production (362.23 mg/L), a 2.39-fold of the control group (Fig. 4D). Hence, the live bacterium was selected an effective elicitor to stimulate PQ production in the subsequent mycelium cultures.

### Effect of live SB1 on fungal morphology and growth

**Fig. 5:**
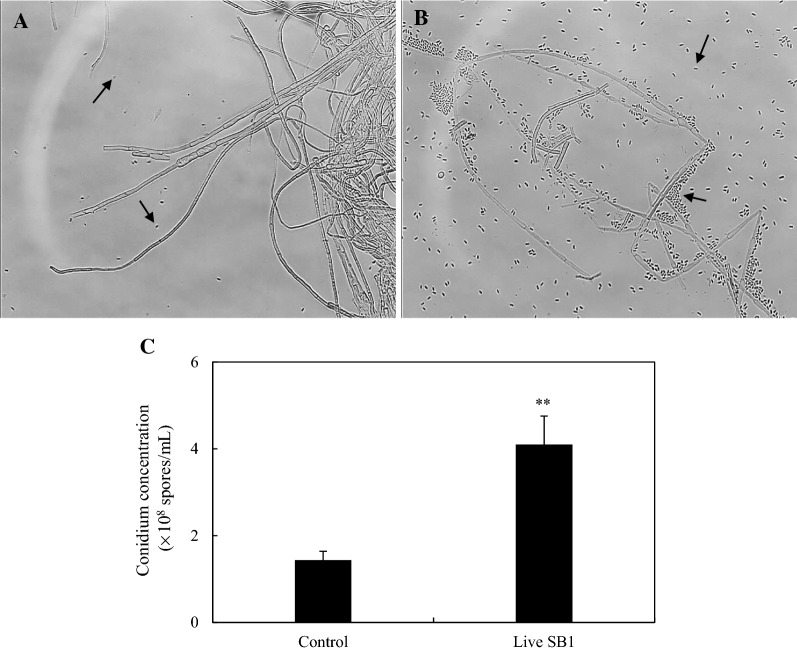
The effect of live *P. fulva* SB1 on the morphologic characteristics of *Shiraia* sp. S9 (×400). The mycelia of S9 were kept on PDA (**A**) without or (**B**) with SB1 cells for 8 days. Arrow indicates the conidium. **C** Effect of live SB1 on the generation of conidium of S9 on day 8. The procedure of fungal–bacterial confrontation assay was the same as specified in Fig. 1. Values are mean ± SD from three independent experiments. **p < 0.01 versus control group

**Fig. 6:**
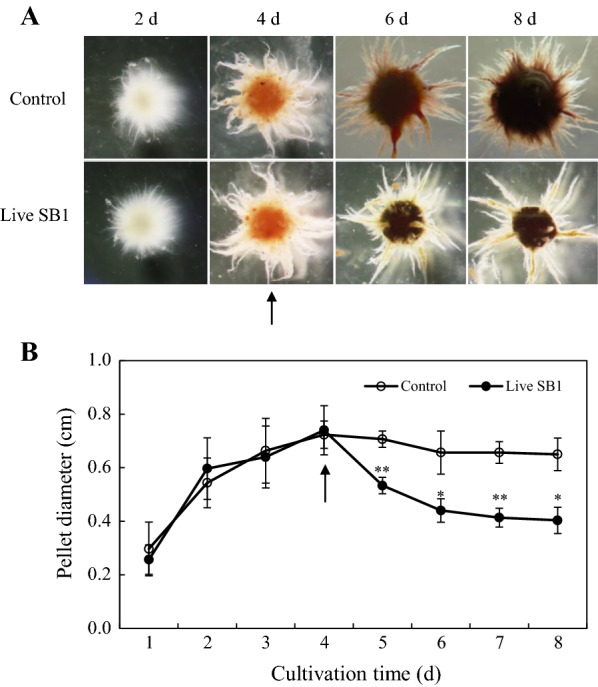
Effect of live *P. fulva* SB1 treatment on mycelium pellets of *Shiraia* sp. S9. **A** Morphology of the pellets at different cultivation time (2, 4, 6 and 8 days) (×80). **B** Time profiles of average pellet diameters during the culture. Pellet diameters were measured in triplicates (50 pellets per replicate). The procedure of fungal–bacterial co-cultures was the same as specified in Fig. 2. The arrow represents the time of addition of live SB1. Values are mean ± SD from three independent experiments. *p < 0.05 and **p < 0.01 versus control

**Fig. 7:**
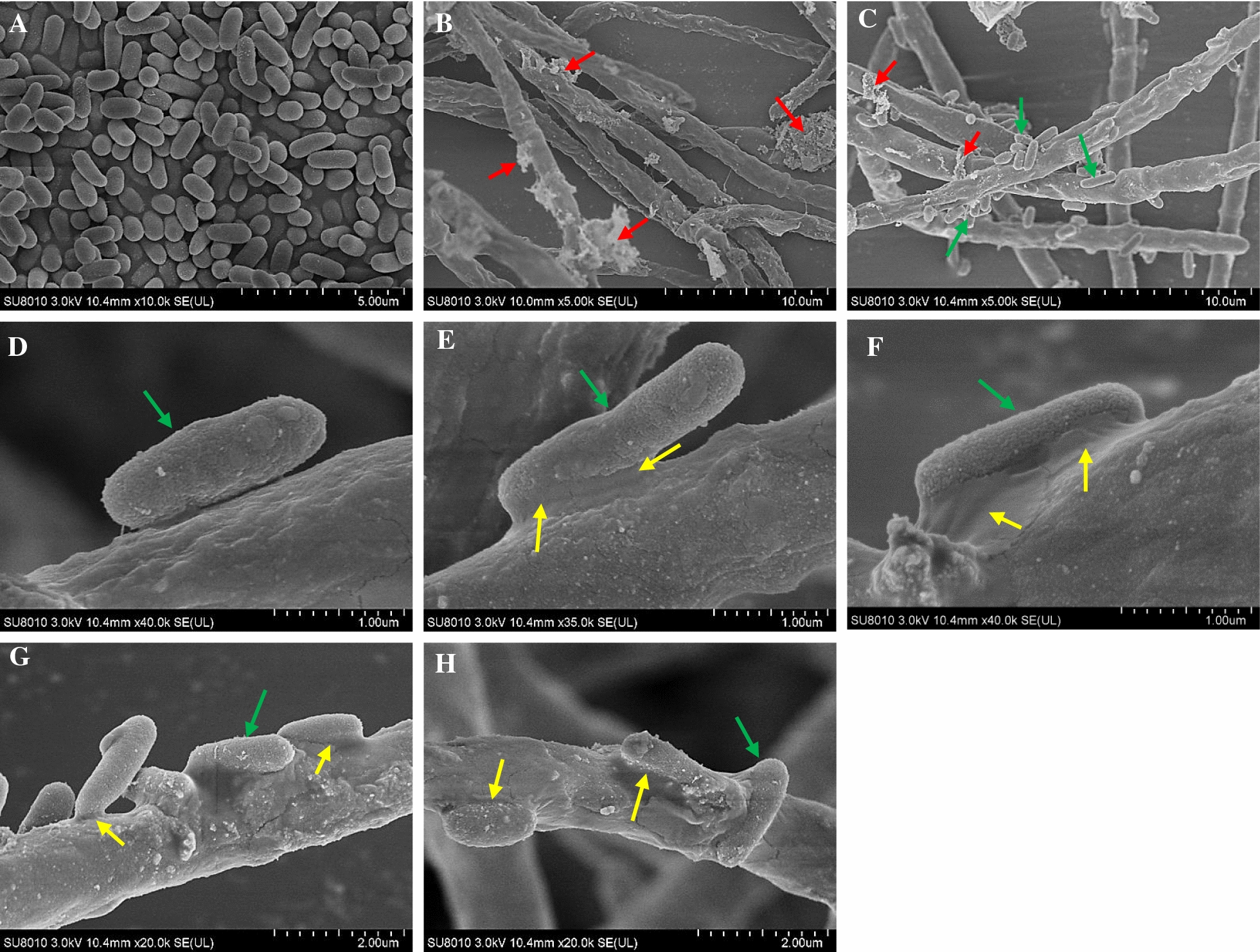
The SEM images of the bacterial–fungal physical attachment. The axenic samples were collected from bacterium *P. fulva* SB1 culture (**A** 12 h) and *Shiraia* sp. S9 culture (**B** 4 days). The mixed samples were collected from the co-cultures at different cultivation time (**C**, **D** 2 h, **E** 12 h, **F** 24 h, **G** 36 h and **H** 48 h). The fungus S9 treated by live SB1 at 200 cells/mL on day 4. Arrow (red) indicates the flocculent structure. Arrow (green) indicates the bacterium SB1. Arrow (yellow) indicates the biofilm

The fungal colonies revealed long and septate hyphae on PDA plates (Fig. 5A and Additional file 1: Figure S4). After the co-cultivation with the bacterium SB1, the majority of S9 mycelia became fragmented and thin markedly (Fig. 5B and Additional file 1: Figure S4). Simultaneously, fungal conidiation was activated largely. The concentration of conidia increased significantly from 1.43 to 4.10 × 10^8^ spores/mL (Fig. 5C). Compared to the fluffier fungal pellets in control group, the smaller and more compact pellets emerged after the addition of the bacterium (Fig. 6A). The pellet diameter was smaller apparently in co-cultures, decreased by 24.53–37.95%, compared to that in the axenic culture from day 5 to 8 (Fig. 6B). In an axenic culture, the rod-shaped SB1 cells maintained their normal morphology (Fig. 7A). However, a relatively small number of floccus-like structure on the surface of S9 hyphae were observed after 2 h of the co-culture on day 4 (Fig. 7B, C). During the early co-culture process (2–12 h), the bacterium cells were observed to have physical contact to the fungus with a thin cell layer along the surface of hyphae (Fig. 7D, E). After 12 h, most of SB1 cells attached to the fungal surface completely and the cell layer became thicker (Fig. 7F, G), whereas some of the bacterium cells were almost merged with fungal hyphae (Fig. 7H).

### Optimization of the co-cultures for enhanced HA production

**Fig. 8:**
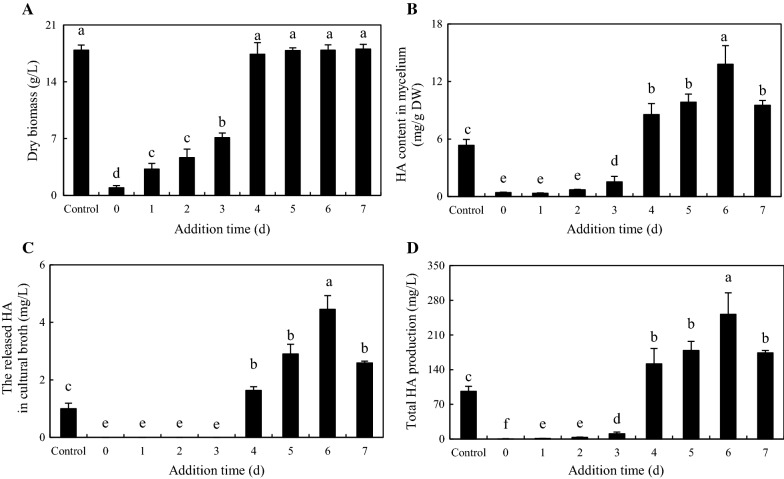
Effects of addition time of live *P. fulva* SB1 on fungal biomass (**A**), HA content in mycelium (**B**), the released HA in cultural broth (**C**) and total HA production (**D**) in submerged culture of *Shiraia* sp. S9. Total HA production refers to the sum of the intracellular and extracellular HA. Values are mean ± SD from three independent experiments. Different letters above the bars mean significant differences (*p* < 0.05)

**Fig. 9:**
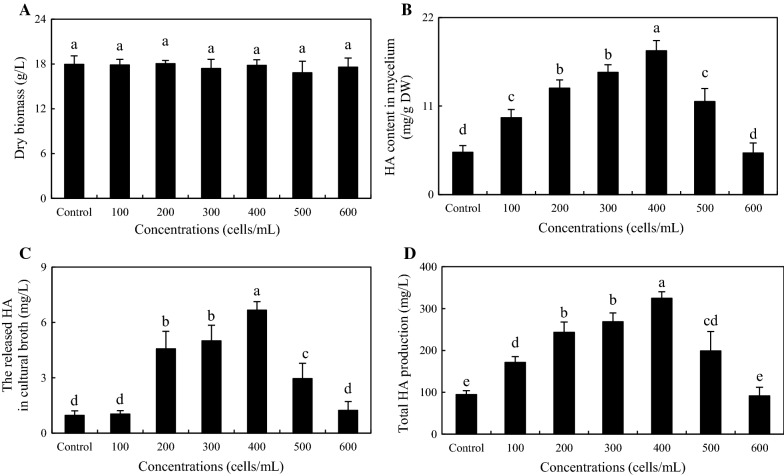
Effects of live *P. fulva* SB1 treatment at different concentrations on fungal biomass (**A**), HA content in mycelium (**B**), the released HA in cultural broth (**C**) and total HA production (**D**) in submerged culture of *Shiraia* sp. S9. Total HA production refers to the sum of the intracellular and extracellular HA. Values are mean ± SD from three independent experiments. Different letters above the bars mean significant differences (*p* < 0.05)

**Fig. 10:**
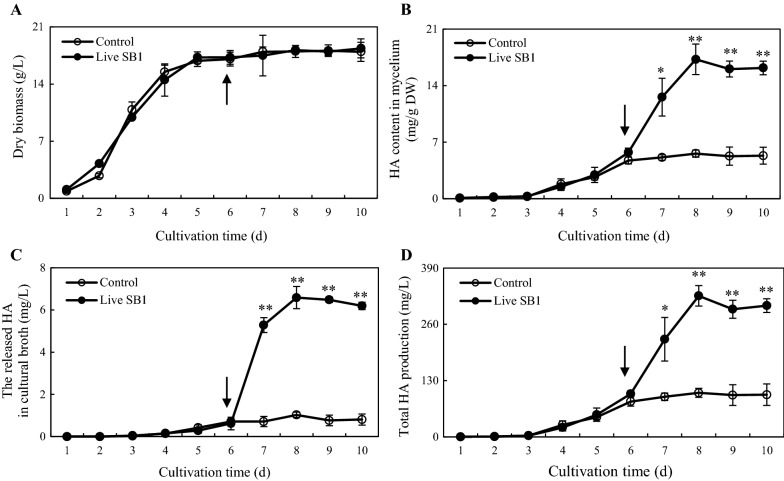
Time profiles of mycelium dry biomass (**A**), HA content in mycelium (**B**), the released HA in cultural broth (**C**) and total HA production (**D**) in submerged culture of *Shiraia* sp. S9 with live *P. fulva* SB1 treatment at 400 cells/mL on day 6. The arrow represents the addition time of live SB1. Total HA production refers to the sum of the intracellular and extracellular HA. Values are mean ± SD from three independent experiments. *p < 0.05 and **p < 0.01 versus control

To optimize the conditions for the co-culture, we investigated the effects of adding time and bacterial concentration on HA production. When the bacterium SB1 was added at the early stage (day 0–3), both the mycelium biomass and HA biosynthesis were suppressed severely by SB1 addition (Fig. 8). However, live SB1 treatment on day 4–7 of initial fermentation have no clear impacts on fungal growth (Fig. 8A), whereas the highest HA contents in both mycelium (13.79 mg/g DW in Fig. 8B) and medium (4.46 mg/L in Fig. 8C) were achieved under the treatment of SB1 on day 6. To further optimize the co-cultures, different concentrations of bacterium SB1 (100–600 cells/mL) were applied to the fungal cultures on day 6. The SB1 at a density of 100–500 cells/mL showed no retardation of fungal growth but promotion of HA production (Fig. 9A, B). When SB1 at a density of 400 cells/mL was added on day 6 of initial fermentation, it promoted not only the accumulation of HA in hyphae (17.86 mg/g DW in Fig. 9B), but also the released HA into the broth at 6.66 mg/L (Fig. 9C) with the maximum total HA production on day 8 (Fig. 9D). Hence, after 2-day treatment of SB1 at 400 cells/mL, total HA production in cultures was enhanced to its peak value 325.87 mg/L, about 3.20-fold of that of control group (Fig. 10).

### Effect of live SB1 on expression of PQ biosynthetic genes

**Fig. 11:**
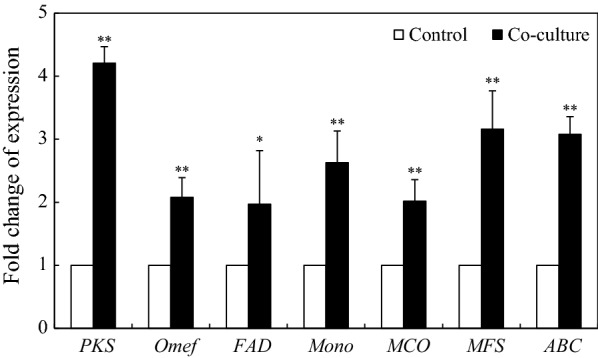
Effect of live *P. fulva* SB1 on the expression of PQ biosynthetic genes of *Shiraia* sp. S9. SB1 was added at 400 cells/mL on day 6 for 2-day treatment. The S9 treated with an equal volume of sterile LB broth instead of bacterial suspension was used as control group. *PKS* polyketide synthase, *Omef O*-methyltransferase, *FAD* FAD/FMN-dependent oxidoreductase, *Mono* monooxygenase, *MCO* multicopper oxidase, *MFS* major facilitator superfamily, *ABC* ATP-binding cassette transporter. Values are mean ± SD from three independent experiments. *p < 0.05, **p < 0.01 versus control group

To investigate the regulation mechanism of live SB1 on PQ production, seven PQ biosynthesis-related unigenes (accession number SRP 151186, http://www.ncbi.nlm.nih.gov/geo/) including polyketide synthase (*PKS*, CL954Contig1), *O*-methyltransferase (*Omef*, CL6443Contig1), FAD/FMN-dependent oxidoreductase (*FAD*, CL2000Contig1), monooxygenase (*Mono*, CL1046Contig1), multicopper oxidase (*MCO*, CL4891Contig1), major facilitator superfamily (*MFS*, CL13Contig3) and ATP-binding cassette transporter (*ABC*, CL1803Contig1) were analyzed by using qRT-PCR after 48 h of the co-culture. The expression of those PQ biosynthesis-related unigenes were up-regulated by SB1 in the co-culture, about 4.2-, 2.1-, 2.0-, 2.6-, 2.0-, 3.2- and 3.1-fold of the mono-culture control, respectively (Fig. 11).

## Discussion

Owing to the unique structures and the conspicuous biological activities of the PQs such as anticancer [2, 23], antiviral [24] and antimicrobial activities [25], biotechnological production of the PQs using mycelium cultures of *Shiraia* sp. S9 is of great practical value. As the lower yield of PQs in mycelium cultures is the major obstruction on industrial production, many bioprocess strategies have been applied in the mycelium cultures to enhance PQ production, including the cultural medium optimization [26] and addition of a surfactant Triton X-100 [13]. In our present study, co-culturing of *Shiraia* sp. S9 and a bacterium *P. fulva* SB1 resulted in not only significant enhancement of PQ biosynthesis but also the excretion of those PQs (Table 1). After our optimization on the conditions for the co-culture, a higher production of HA (325.87 mg/L) was achieved to 3.20-fold of that in axenic *Shiraia* culture (Fig. 10). It has been reported previously that higher HA yield under the elicitation of a repeated ultrasound [27], light–dark shift [28] and red light [29] was from 175.53 to 247.67 mg/L. Compared with these elicitation techniques, the established co-culture is of great potential to be a novel strategy for the biotechnological production for HA production.

Bacterium–fungus interaction has been utilized in inducing previously undescribed fungal metabolites or improving constitutively present secondary metabolites. The addition of a marine bacterium CNJ-328 could induce the biosynthesis of new pimarane diterpenoids, libertellenones A–D in cultures of *Libertella* sp. CNL-523 [30]. An enhanced production (up to 78-fold) of known metabolites and three new natural products were identified only in co-cultures of endophytic *Fusarium tricinctum* and the bacterium *Bacillus subtilis* 168 trpC2 [31]. Although fungal fruiting bodies harbor rich and complex microbial communities, the role of the associated bacteria on the biosynthesis of fungal secondary metabolites is not well understood. In our present study, a bacterium *P. fulva* SB1 isolated from *Shiraia* fruiting body could elicit both PQ accumulation in hyphal cells and the excretion to the medium (Table 1 and Fig. 2). PQ biosynthesis has been shown to start from acetyl CoA-malonyl CoA condensation and decarboxylation reactions catalyzed by polyketide synthase of *S. bambusicola* (*SbaPKS*) [32, 33]. Similar to other fungal non-reducing *PKSs* [34], *SbaPKS* contains typical perylenequinone domains including an acyl-carrier protein (ACP), ACP transacylase (SAT), a ketosynthase (KS), a malonyl-CoA: ACP transacylase (MAT), product template (PT), and a thioesterase/Claisen-like cyclase (TE/CLC) domain. In our present study, the up-regulation of *Shiraia PKS* expression by live bacterium SB1 was validated using qRT-PCR (Fig. 11). The result was consistent with those found in *Aspergillus nidulans* in response to challenge by the bacterium *Streptomyces hygroscopicus* [35], suggesting that *PKS* may be target gene for the activation of the bacterium on fungal metabolites. It was reported that *SbaPKS* had a significant regulation role on expression of adjacent genes in HA biosynthesis gene cluster such as *O*-methyltransferase/FAD-dependent monooxygenase (*Mono*), *O*-methyltransferase (*Omef*), FAD/FMN-dependent oxidoreductase (*FAD*), multicopper oxidase (*MCO*) and major facilitator superfamily transporter (*MFS*) gene [33]. In this study, the bacterial treatment also up-regulated significantly the expression of these adjacent genes in charge of the polyketide oxidations, hydrations and methyl modification (Fig. 11), suggesting that the whole gene cluster for PQ biosynthesis could be activated by the live bacterium. It was interesting to find that the bacterium enhance the transcript levels of MFS and ABC transporter genes (Fig. 11). Both of them were reported to be involved in hypocrellin secretion [36, 37]. Hence, the enhancement of the extracellular PQ production in the co-cultivation (Table 1 and Fig. 2) may be due to the up-regulation of both *MFS* and *ABC*.

To investigate the mechanism of co-cultures of *Shiraia* and *P. fulva* SB1 for enhanced PQ production, we treated *Shiraia* mycelium culture with live bacterium SB1, bacterial extracts (BE and BPS) and the dialysed bacterial cells (Fig. 4). Our results showed that the live bacterium was the best elicitor to stimulate PQ production among these treatments. The direct contact between the bacterium and the fungus was also confirmed by the scanning electron microscopical observation (Fig. 7). Simultaneously, our results showed the diffusible low molecular weight molecules (< 8–14 kDa) and the bacterial extracts (BE and BPS) could yield a lower but significant enhancement of PQ production (Fig. 4). These results suggested that not only intimate physical interactions but some chemical signals could contribute to the bacterial–fungal communication leading to the transcript induction of PQ biosynthetic genes. On the other hand, our results showed a stimulatory effect of living bacterial cells of *P. fulva* SB1 on the mass production of *Shiraia* conidia (Fig. 5). A similar bacterial effect has previously been observed for the sporulation and germination of *Phytophthora alni* [38]. Varese et al. [19] found *Pseudomonas* from fruiting bodies of *Suillus grevillei* could enhance the growth of its mycobiont. In our present study, although the mycelial biomass was not altered by the live bacterium, the fungal pellet diameters became smaller and more compact in the co-culture (Fig. 6). Since smaller pellets are usually beneficial to substrate absorption and oxygen metastasis for fungal metabolite production in the liquid cultures, we applied a low intensity ultrasound (0.28 W/cm^2^ at 40 kHz) or a light/dark shift (24:24 h) to mycelium cultures to induce smaller *Shiraia* pellets in our previous reports [27, 28]. In current work we presented a possible control on fungal pellets by adding the live bacterium in mycelium cultures. The correlation between fungal morphology and metabolite production in co-cultures needed to be further elucidated for understanding the role of live bacterium in bacterial–fungal interactions.

## Conclusion

Although fungal fruiting bodies contain diverse microbial communities, the regulation on metabolites of host fungi and physiological responses induced by the associated bacteria have not been well studied. To the best of our knowledge, this study is the first to demonstrate that a fruiting body-associated bacterium could regulate metabolite production (PQs) of the host fungi. Our study clearly showed the elicitation of the bacterium *P. fulva* SB1 was strongly dependent on direct contact between the bacterial and fungal mycelia as well as BPS and small metabolites (< 10 kD). The live bacterium didn’t only participate in the transcript induction of PQ biosynthetic genes, but also up-regulated *MFS* and *ABC* expression for PQ exudation. Although the detailed mechanisms for bacterial signal recognition and transduction need to be further investigated, our study provided a new effective co-culture strategy for PQ production of *S. bambusicola*. More new bioactive PQs are expected to be discovered in the established co-cultures in our further studies.

## Materials and methods

### Strains and culture conditions

The fruiting body associated bacteria and the hosting fungus *Shiraia* were isolated from 15 fruiting bodies (5 fruiting bodies per replicate) collected from bamboo (*Brachystachyum densiflorum*) shoots from June to July, 2016 in Tianmu Mountain of Hangzhou, Zhejiang, China [39]. The associated bacteria were isolated by using surface sterilization method [40], and successively transferred and re-streaked until pure cultures were obtained. Finally, a total of 31 bacterial strains were isolated from the fresh *Shiraia* fruiting bodies. The PQ-producing strain *Shiraia* sp. S9 (CGMCC16369) was isolated from fresh *Shiraia* fruiting bodies. The S9 strain was cultured routinely and stored at 4 °C on potato dextrose agar (PDA) slant culture. To initiate the cultures, the S9 strain was cultivated on fresh PDA medium in a Petri dish (10 cm diameter) at 28 °C for 8 days. More details on the preparation of seed culture, components of the liquid medium and culture conditions were given in our previous report [27]. The S9 seed culture (5 mL) was added into a 150-mL Erlenmeyer flask containing 50-mL liquid medium and the culture was incubated for 8–10 days at 28 °C in a rotary shaker at 150 rpm.

### Fungal–bacterial confrontation assay

An in vitro confrontation bioassay between the hosting fungus S9 and the associated bacteria was conducted according to the method reported by Wang et al. [41]. In brief, S9 strain was maintained on PDA medium in a Petri dish at 28 °C for 8 days. Then, a small piece (5 mm × 5 mm) of marginal mycelium with agar was cut and placed in the center of 10-cm PDA plate for 4 days. To analyze the effect of live bacterial isolates on mycelia growth and PQ secretion of S9 strain preliminarily, the single colony of bacterium was inoculated in LB broth (without agar) at 37 °C on a rotary shaker at 200 rpm for 12 h. Then bacterial suspension (10 µL) was streaked in two parallel straight lines, approximately 7 cm apart from each other (Fig. 1A). After incubation for 8 days, the S9 colony morphology was observed and photographed. The S9 strain treated with an equal volume of sterile LB broth instead of bacterial suspension was used as control group.

### Identification and characterization of active bacterium

After the fungus–bacteria confrontation assay, No. 11 strain showed the most significant stimulation effect on the accumulation of red fungal pigments (Additional file 1: Figure S1) and total PQ accumulation in PDA medium (Additional file 1: Table S1), which was described in more detail in section of ‘Results’. The total DNA of the isolate (No. 11) was extracted from the overnight bacterial culture using Plant Genomic DNA Kit (Tiangen, Beijing, China) and DNA integrity and purity were detected using the Agilent 2100 Bioanalyzer (Agilent Technologies, CA, USA). The 16S rDNA was amplified using the conserved bacterium-specific primers (27F/1492R) and PCR reaction [42], and then a phylogenetic tree of the bacterium was constructed by the Neighbor-joining method using molecular evolutionary genetics analysis (MEGA7) [43]. The essential biochemical characteristics such as Gram character and gelatin liquefaction of the bacterium have also been analyzed [44].

### Co-cultivation of *Shiraia* sp. S9 with the bacterium

The seed culture (5 mL) of fungal strain S9 was added into a 150-mL Erlenmeyer flask containing 50-mL liquid medium and the culture was kept at 28 °C in a rotary shaker at 150 rpm [27]. The single colony (approximately 2 mm diameter) of bacterium SB1 was inoculated in LB broth at 37 °C on a rotary shaker at 200 rpm for 12 h. Then bacterial cells were transferred into the submerged culture of S9 at a final density of 100–600 cells/mL at different stages of S9 growth (0–7 days). As a control, equivalent volume of fresh LB broth was transferred into the submerged culture of S9. After the co-cultures at 28 °C on a rotary shaker at 150 rpm, the mycelia were harvested and dried to constant weight in 60 °C oven to evaluate the fungal biomass and PQ contents.

In addition to live bacteria, bacterial extracts and bacteria inside dialysis tubing were also tested of their effects on fungal PQ production in the cultures. For preparation of the bacterial extracts, cells were harvested from 12 h—culture of bacterium SB1 in LB broth by centrifugation at 12,000 rpm for 10 min. The cell mass (5 g, fresh weight, FW) was re-suspended in distilled water (50 mL) and autoclaved at 121 °C for 25 min. The autoclaved supernatant was collected as the bacterial extract (BE) by centrifugation at 6000 rpm for 10 min. To prepare crude bacterial polysaccharide, BE was mixed with threefold volume of 95% ethanol and maintained at 4 °C until it was precipitated completely. The freeze-dried precipitate was collected as crude bacterial polysaccharide (BPS). In addition, total sugar content of BPS fractions was determined by the anthrone–sulfuric acid method [45] and total protein content was measured by using Enhanced BCA Assay Kit (Beyotime, Nanjing, China) according to the manufacturer’s protocol (Additional file 1: Table S3). Finally, a non-contact co-culture was set up [46] by placing 2 mL live bacteria at a density of 5 × 10^3^ cells/mL inside a dialysis tubing (1 × 5 cm^2^, molecular weight cut-off 8–14 kDa, Sinopharm, Nanjing, China) and immersed dialysis tubing in 50-mL liquid medium in a 150-mL Erlenmeyer flask with 4-day-old cultures of fungal S9. To investigate their effects on PQ production, the live bacteria SB1 (200 cells/mL, final density in flask), BE (25–600 mg/L), crude BPS (25–600 mg/L) and the dialysis tubing with the live bacteria were added separately into the mycelium cultures of S9 on the same time (day 4). Then, the cultures were maintained on a rotary shaker at 150 rpm at 28 °C for 4 days. The S9 strain treated with the equal volumes of sterile LB broth as control group.

### Microscopic morphology observation

To observe the sporulation and aerial mycelium formation of *Shiraia* sp. S9, the fungal colony from 8-day PDA plate for confrontation assay was eluted with 5-mL distilled water. Then, the morphological characteristics were observed using a light microscope (CKX41, Olympus, Tokyo, Japan) and photographed using the differential interference contrast (DIC) optics system (Leica, Milton Keynes, UK).

To analyze the influence of live bacterium on fungal pellets at different cultivation time (2, 4, 6 and 8 days), the pellets were viewed by a stereoscopic microscope (SMZ1000, Nikon, Tokyo, Japan) and photographed using an external camera (Coolpix S4, Nikon, Japan). Meanwhile, the pellet diameters were calculated in triplicates (50 objects in a replicate) on day 1–10.

To investigate the interaction between the bacterium and its host fungus, the physical attachment of the interaction between S9 and SB1 was observed using scanning electron microscope (SEM, S-4700, Hitachi, Japan) according to the methods described by Wang et al. [47]. The samples were collected from different time point (2, 12, 24, 36 and 48 h) and fixed with 2.5% glutaraldehyde for SEM observation.

### PQ extraction and quantification

The PQs in PDA plates, mycelia and cultural broth were extracted according to the previously described method [12]. The individual PQs were measured by a reverse-phase Agilent 1260 HPLC system (Agilent Co., Wilmington, USA) equipped with the Agilent HC-C18 column (250 × 4.6 mm dimension) with a mobile phase (acetonitrile: water at 65: 35, v/v) at 1 mL/min for 20 min and with UV detection at 465 nm [10]. The sum of HA, HB, HC, EA, EB and EC was taken as the total PQ contents.

### Quantitative real-time PCR analysis

After 48 h—coculture with live bacterium, total RNA of the fungal mycelia was extracted using RNAprep pure Plant Kit (Tiangen, Beijing, China). The primers of target genes (Additional file 1: Table S4) for PQ biosynthesis and internal reference gene (18S ribosomal RNA) were designed with the Primer Express software (Applied Biosystems, Foster City, USA). The qRT-PCR condition and procedure was set and performed as our previous report [27].

### Statistical analysis

All treatments consisted of triplicate independent repeats (ten plates or flasks per replicate). Student’s t-test and one-way analysis of variance (ANOVA) with Dunnett’s multiple comparison tests were applied for experimental results. All results are expressed as mean ± standard deviation (SD) and *p* < 0.05 being considered statistically significant.

## Highlights

- A perylenequinonoid (PQ)-producing fungus *Shiraia* sp. S9 was isolated from the fruiting body.
- A fruiting body associated bacterium *Pseudomonas fulva* SB1 was screened to elicit fungal PQ biosynthesis.
- Both PQ in mycelia and its release in medium were enhanced by the live bacterium.
- The highest HA production (325.87 mg/L) was achieved after 2 days of the co-culture.
- The bacterium SB1 participated in the transcript induction for PQ biosynthesis and exudation.

## Additional file

**Additional file 1: Table S1.** Effect of live bacteria on total PQ accumulation of host fungus *Shiraia* sp. S9 in solid-state cultures. **Table S2.** Physiological and biochemical characteristics of bacterium No. 11 named *P. fulva* SB1. **Table S3.** Total sugar and protein contents of various fractions of bacterium SB1 extract. **Table S4.** The primers of the target genes and the internal reference gene used for qRT-PCR. **Figure S1.** The examples of the effects of live bacteria on the growth and red pigments secretion of *Shiraia* sp. S9. **Figure S2.** Effects of hot water extract (BE) of P. fulva SB1 cells at different concentrations on mycelium dry biomass (A), PQ contents in mycelium (B), the released PQ in cultural broth (C) and total PQ production (D) in submerged culture of *Shiraia* sp. S9. **Figure S3.** Effects of crude polysaccharide (BPS) of *P. fulva* SB1 cells at different concentrations on mycelium dry biomass (A), PQ contents in mycelium (B), the released PQ in cultural broth (C) and total PQ production (D) in submerged culture of *Shiraia* sp. S9. **Figure S4.** The effect of live *P. fulva* SB1 on conidia production of *Shiraia* sp. S9 (400 ×). The mycelia of S9 were kept on PDA **(A**, **C**, **E)** without or (**B**, **D**, **F)** with SB1 cells for 8 days. *Arrow* indicates the conidium. The procedure of fungal–bacterial confrontation assay was the same as specified in Fig. 1.

## Data Availability

All data generated or analyzed during this study are included in this published article and its Additional file.
