# Supplementary material for: Inducing perylenequinone production from a bambusicolous fungus Shiraia sp. S9 through co-culture with a fruiting body-associated bacterium Pseudomonas fulva SB1
Source: Microb Cell Fact. 2019 Jul 5;18:121. doi: 10.1186/s12934-019-1170-5 (PMC6612088; doi:10.1186/s12934-019-1170-5)
Supplement: Supplementary file 1 — Additional file 1: Table S1. Effect of live bacteria on total PQ accumulation of host fungus Shiraia sp. S9 in solid-state cultures. Table S2. Physiological and biochemical characteristics of bacterium No. 11 named P. fulva SB1. Table S3. Total sugar and protein contents of various fractions of bacterium SB1 extract. Table S4. The primers of the target genes and the internal reference gene used for qRT-PCR. Figure S1. The examples of the effects of live bacteria on the growth and red pigments secretion of Shiraia sp. S9. Figure S2. Effects of hot water extract (BE) of P. fulva SB1 cells at different concentrations on mycelium dry biomass (A), PQ contents in mycelium (B), the released PQ in cultural broth (C) and total PQ production (D) in submerged culture of Shiraia sp. S9. Figure S3. Effects of crude polysaccharide (BPS) of P. fulva SB1 cells at different concentrations on mycelium dry biomass (A), PQ contents in mycelium (B), the released PQ in cultural broth (C) and total PQ production (D) in submerged culture of Shiraia sp. S9. Figure S4. The effect of live P. fulva SB1 on conidia production of Shiraia sp. S9 (400 ×). The mycelia of S9 were kept on PDA (A, C, E) without or (B, D, F) with SB1 cells for 8 days. Arrow indicates the conidium. The procedure of fungal–bacterial confrontation assay was the same as specified in Fig. 1. [file 12934_2019_1170_MOESM1_ESM.pdf]

Additional file 1

Inducing perylenequinone production from a bambusicolous fungus *Shiraia* sp. S9 through co-culture with a fruiting body-associated bacterium *Pseudomonas fulva* SB1

Yan Jun Ma, Li Ping Zheng, Jian Wen Wang

✉ Jian Wen Wang
jwwang@suda.edu.cn; bcjwwang@gmail.com

Table S1

Effect of live bacteria on total PQ accumulation of host fungus *Shiraia* sp. S9 in solid-state cultures.

Strain No.	Total PQ contents (mg/cm^2)
Control	3.94 ± 0.16
1	ND
2	1.60 ± 0.08**
3	2.17 ± 0.15**
4	1.34 ± 0.04**
5	1.81 ± 0.05**
6	3.62 ± 0.33
7	1.99 ± 0.16**
8	4.90 ±0.10*
9	1.79 ± 0.30*
10	2.10 ±0.03**
11	9.21 ± 0.21**
12	5.05 ± 0.19**
13	4.10 ± 0.07
14	3.78 ± 0.21
15	ND
16	1.55 ± 0.06**
17	1.26 ± 0.16**
18	ND
19	ND
20	5.13 ± 0.21**
21	4.24 ± 0.27
22	ND
23	4.75 ± 0.05*
24	4.99 ± 0.11**
25	3.86 ± 0.15
26	1.27 ± 0.05**
27	1.70 ± 0.18**
28	4.16 ± 0.28
29	2.79 ± 0.17*
30	3.98 ± 0.20
31	1.10 ± 0.03**

Values are mean ± SD from three independent experiments. *$p < 0.05$, **$p < 0.01$ versus control group. ND means not detected.

Table S2

Physiological and biochemical characteristics of bacterium No.11 named *P. fulva* SB1.

Strain	Gram stain	Glucose utilization	Starch hydrolysis	Citrate utilization	Nitrate reduction	Oxidase test	Catalase test	GL test
P. fulva SB1	-	+	-	+	-	+	+	+

(+) positive reaction; (-) negative reaction; (GL) gelatin liquefaction.

Table S3

Total sugar and protein contents of various fractions of bacterium SB1 extract.

Fractions	Sugar (mass %)	Protein (mass %)
Hot water extract (BE)	30.25 ± 2.06	11.30 ± 1.62
Crude polysaccharide (BPS)	52.13 ± 0.45	27.18 ± 1.19

Values are mean ± SD from three independent experiments.

Table S4

The primers of the target genes and the internal reference gene used for qRT-PCR.

Unigene ID	Gene description	Sequence
18S	Reference gene	F: 5’-ACGCAGCGAAATGCGATAAG-3’<br>R: 5’-CAAATTGTGCTGCGCTCCAA-3’
CL13Contig3	*Shiraia* sp. slf14 major facilitator superfamily (MFS) transporter	F: 5’-TCCATTGTTCCAGGCGTACC-3’<br>R: 5’-TATGCCTCGCCGTTCTTTGT-3’
CL954Contig1	*Shiraia* sp. slf14 polyketide synthase	F: 5’-GCTGTCCTGAAACGACTGGA-3’<br>R: 5’-CGAATATCGGGCACGTCTGA-3’
CL1046Contig1	*Shiraia* sp. slf14 hydroxylase	F: 5’-GGACGATTCCACGCGATTTG-3’<br>R: 5’-CATGGCTGACCGCATCATTG-3’
CL1803Contig1	ATP-binding cassette transporter 1	F: 5’-CATGTCTCCCGACCTCATCG-3’<br>R: 5’-AAGCGCAGTGTTTCGTTGAC-3’
CL2000Contig1	*Shiraia* sp. slf14 FAD/FMA-dependent oxidoreductase	F: 5’-GATGGTTGCGTTGGCAAGTT-3’<br>R: 5’-GCTTCCCACCCATACGACAA-3’
CL4891Contig1	Multicopper oxidase	F: 5’-CCCATCAAACCATTCGTCGC-3’<br>R: 5’-ATTTTGTGGCCGAGGTCCAT-3’
CL6443Contig1	*Shiraia* sp. slf14 *O*-methyltransferase	F: 5’-GCTGGTGGACCTCTCCTTTC-3’<br>R: 5’-AGCGTGCCTTCAGGTAGTTC-3’

F: forward primer, R: reverse primer.

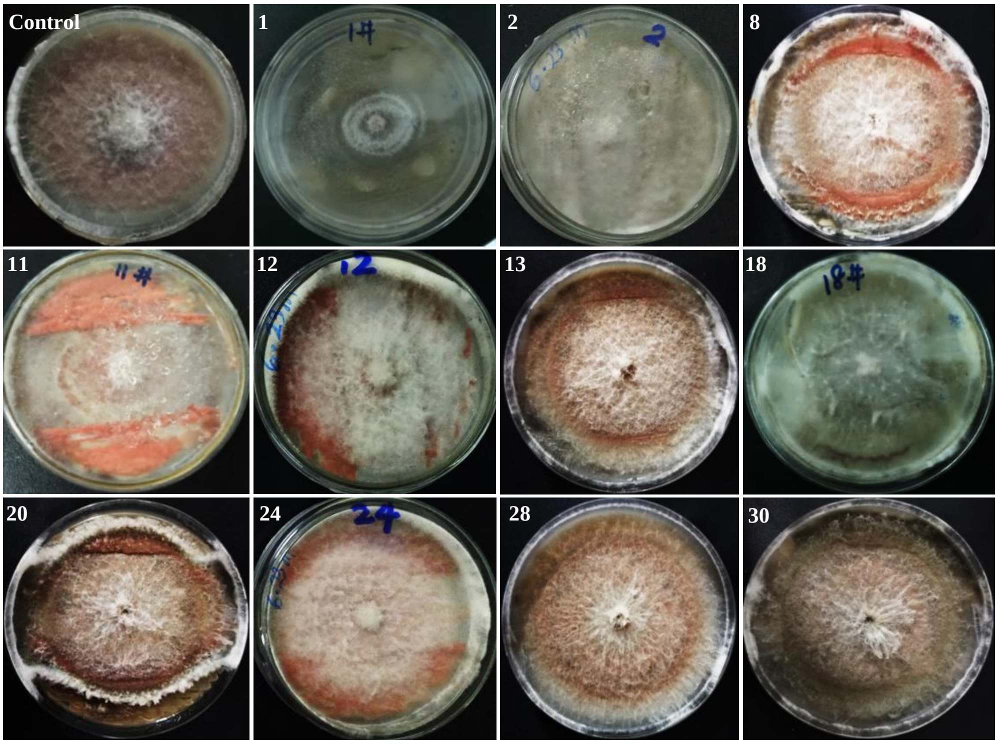

Fig. S1 The examples of the effects of live on the growth and red pigments secretion of *Shiraia* sp. S9. The procedure of fungal-bacterial confrontation assay was the same as specified in Fig. 1.

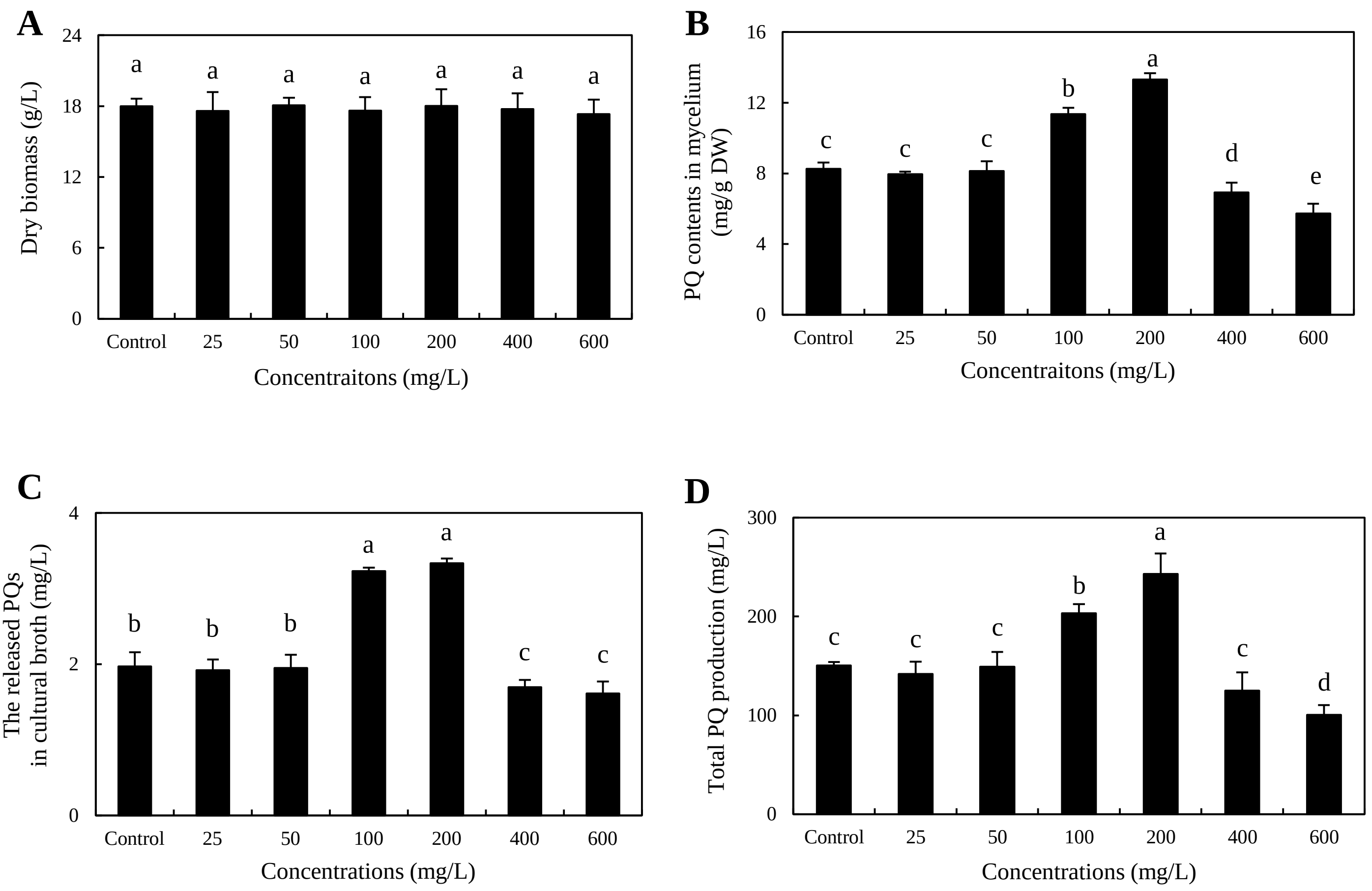

Fig. S2 Effects of hot water extract (BE) of *P. fulva* SB1 cells at different concentrations on mycelium dry biomass (**A)**, PQ contents in mycelium (**B**), the released PQ in cultural broth (**C**) and total PQ production (**D)** in submerged culture of *Shiraia* sp. S9. The procedure was the same as specified in Fig. 2. Total PQ production refers to the sum of the intracellular and extracellular PQs. Values are mean ± SD from three independent experiments. Different *letters* above the bars mean significant differences ($p < 0.05$).

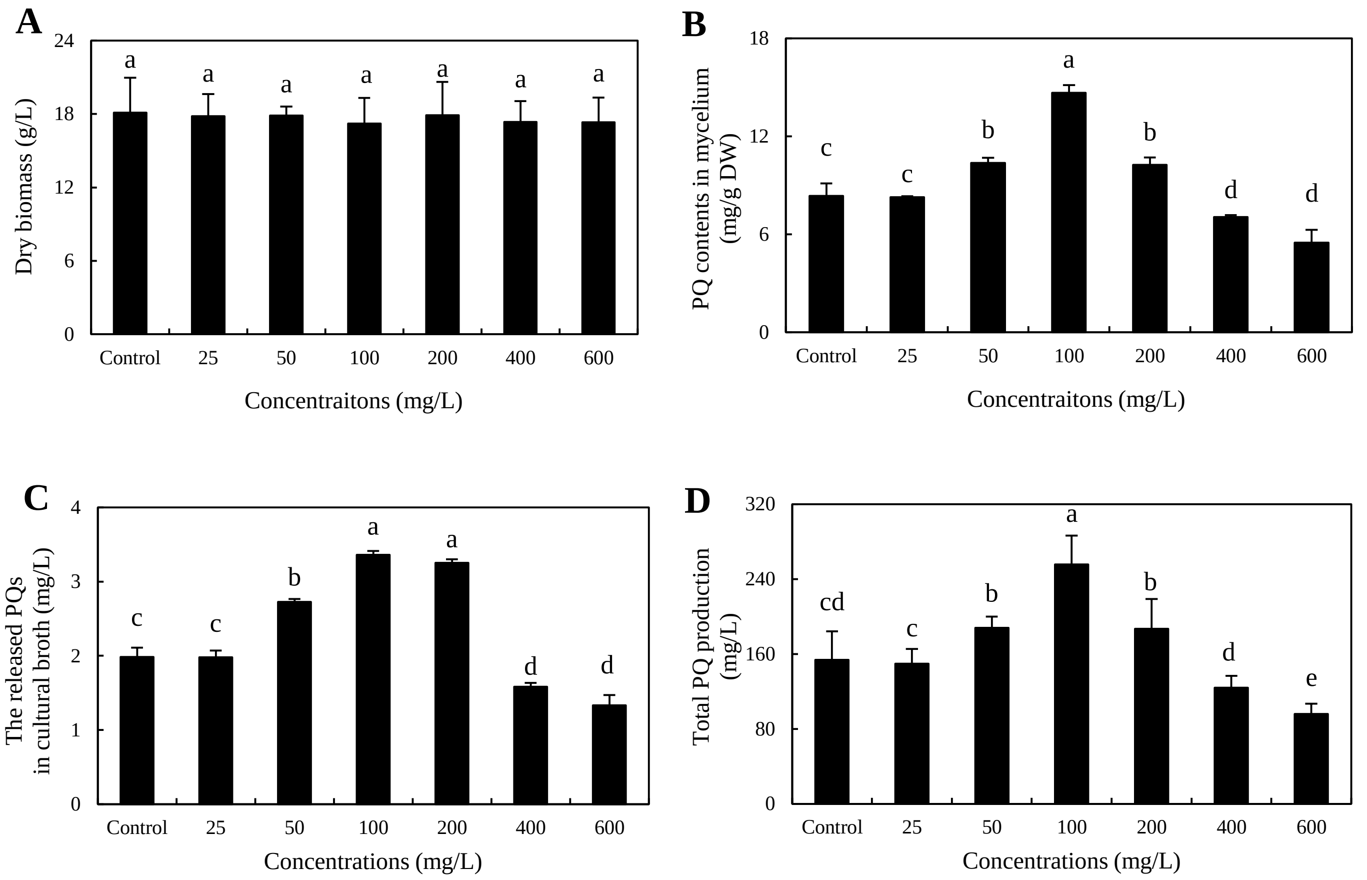

Fig. S3 Effects of crude polysaccharide (BPS) of *P. fulva* SB1 cells at different concentrations on mycelium dry biomass (**A**), PQ contents in mycelium (**B**), the released PQ in cultural broth (**C**) and total PQ production (**D**) in submerged culture of *Shiraia* sp. S9. The procedure was the same as specified in Fig. 2. Total PQ production refers to the sum of the intracellular and extracellular PQs. Values are mean ± SD from three independent experiments. Different *letters* above the bars mean significant differences ($p < 0.05$).

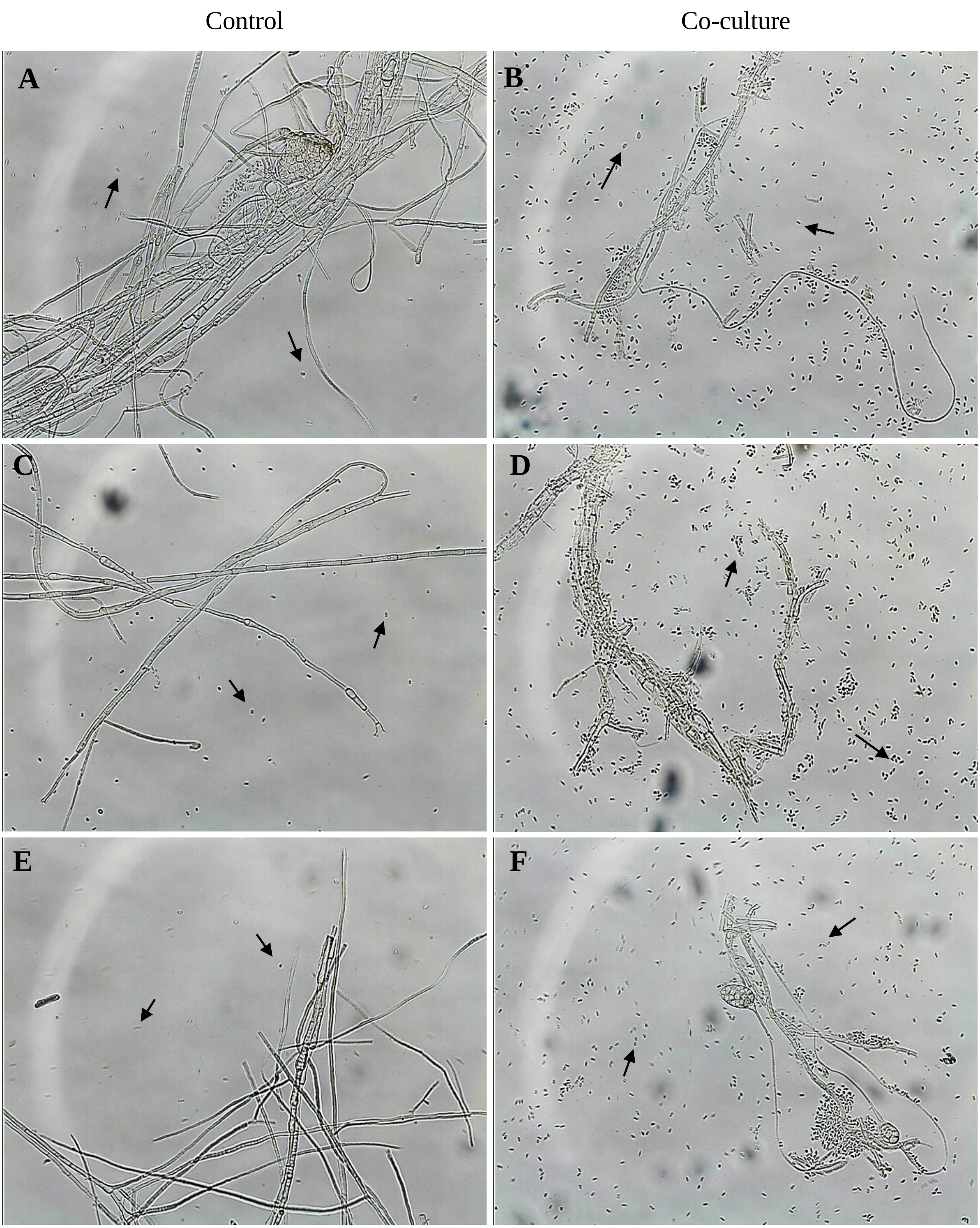

Fig. S4 The effect of live *P. fulva* SB1 on conidia production of *Shiraia* sp. S9 (400 ×). The mycelia of S9 were kept on PDA without **(A, C, E)** or with **(B, D, F)** SB1 cells for 8 days. Arrow indicates the conidium. The procedure of fungal-bacterial confrontation assay was the same as specified in Fig. 1.
